# The entactogen MDMA (3,4-methylenedioxymethamphetamine, “Ecstasy”) disrupts helping behaviour while reinforcing electrophysiological indicators of potentially associated synaptic plasticity in male Sprague-Dawley rats

**DOI:** 10.3389/fphar.2026.1779772

**Published:** 2026-04-17

**Authors:** Patricio Sáez-Briones, Amanda Silva-Rodríguez, Michelle Morales-Vidal, Yaniz Sepúlveda-Fernández, Dorys Jara-Clen, Michelle Castro-Choapa, Pablo Livacic-Rojas, Darío Martínez-Afani, Bruce K. Cassels, Rafael Barra, Luis Constandil, Jeffri Retamal, Alejandro Hernández

**Affiliations:** 1 Laboratory of Neuropharmacology and Behaviour, School of Medicine, Faculty of Medical Sciences, Universidad de Santiago de Chile, Santiago, Chile; 2 School of Psychology, Faculty of Humanities, Universidad de Santiago de Chile, Santiago, Chile; 3 School of Biochemistry, Faculty of Chemistry and Biology, Universidad de Santiago de Chile, Santiago, Chile; 4 Laboratory of Biodynamic Chemistry, Department of Chemistry, Faculty of Sciences, Universidad de Chile, Santiago, Chile; 5 Centre for Biomedical and Applied Research (CIBAP), Faculty of Medical Sciences, Universidad de Santiago de Chile, Santiago, Chile; 6 Laboratory of Neurobiology, Faculty of Chemistry and Biology, Universidad de Santiago de Chile, Santiago, Chile

**Keywords:** 3,4-methylenedioxymethamphetamine, helping behaviour, LTD (long term depression), LTP (long term potentiation), serotonin, oxytocin

## Abstract

**Introduction:**

In humans, empathy is expressed through various prosocial behaviours between individuals that may be enhanced after intake of the synthetic entactogen MDMA (3,4-methylenedioxymethamphetamine, “Ecstasy”) as the behavioural expression of the so-called entactogenic syndrome. Rodents may also exhibit empathy-like behaviours, such as social interaction and helping behaviour. In this regard, while social interaction has been reported to be enhanced by MDMA, the effects of this drug on helping behaviour remain unexplored. Nevertheless, because helping behaviour is considered as part of the prosocial repertoire, it may be hypothesised that MDMA should enhance it.

**Methods:**

In the present study, the evaluation of a subtoxic dose range (0.25 mg/kg, 0.5 mg/kg, 1 mg/kg, 5 mg/kg, and 10 mg/kg i.p.) of MDMA on helping behaviour in adult male rats has been conducted using a standardised behavioural setup based on the intrinsic aversion of these animals to water. In addition, as helping behaviour may require a complex interaction between motivational and higher cognitive processes, the neuroplastic effects of MDMA (10 mg/kg i.p.) on cortical and subcortical loci were studied *in vivo* in anaesthetised rats.

**Results and discussion:**

Behavioural data indicated that 5 mg/kg and 10 mg/kg of MDMA fully suppressed helping behaviour; 1 mg/kg and 0.5 mg/kg induced partial inhibition only after interchanging roles; and 0.25 mg/kg had no effect. The inhibitions observed at the highest doses (5 mg/kg, and 10 mg/kg) were not reversed after interchanging roles. Electrophysiological data showed that MDMA reinforced long-term depression (LTD) elicited in the nucleus accumbens (NAc) core following stimulation of the dorsal raphe nucleus (DRN). In addition, MDMA increased transcallosal-evoked long-term potentiation (LTP) in the anterior cingulate cortex (ACC) in a serotonin (5-HT)- and oxytocin (OXT)-dependent manner. Taken together, these data support the notion that MDMA disrupts helping behaviour, even though the neuroplastic effects elicited by the drug align with the mechanisms described to promote prosocial/empathic behaviours. The results may suggest a negative modulation of MDMA on neural processes that are essential for the execution of helping behaviour without affecting the willingness to help.

## Introduction

Empathy refers to the ability to share emotions (Keysers et al., 2022), which is expressed through prosocial behaviours. Operationally, prosocial behaviours occur because of prior emotional contagion between individuals (Cronin, 2012; Meyza et al., 2017; Sivaselvachandran et al., 2018) and include basic social interaction and eventually other complex interactions between individuals, such as helping behaviours. Indeed, prosocial behaviours, as part of the social interactions’ repertoire, favour the interactions between individuals where the decisions of one individual affecting the state of others may (or may not, see Vieira and Olsson, 2024) be related to empathy (Burkett et al., 2016; Decety et al., 2016) and are associated with the release of oxytocin (OXT) both in humans (Guastella and Hickie, 2016) and in rodents (Burkett et al., 2016). In this regard, the synthetic entactogen MDMA (3,4-methylenedioxymethamphetamine, “Ecstasy”) may enhance social interaction in rodents (Morley and McGregor, 2000; Sáez-Briones et al., 2019), showing only minimal intrinsic rewarding properties when evaluated in the intravenous self-administration paradigm in comparison to stimulants like cocaine (Cornish et al., 2003), resembling the induction of the entactogenic syndrome in humans (Sáez-Briones and Díaz-Véliz, 2012; Sáez-Briones and Hernández, 2013). Interestingly, it has been shown that prosocial behaviour in rats is modulated by oxytocin (OXT) release (Thompson et al., 2007; Burkett et al., 2016). This key event may be triggered by serotonin (5-HT) acting on serotonergic 5-HT_1A_ receptors (Hunt et al., 2011). It has been proposed that MDMA may induce prosocial behaviour as a result of a massive non-exocytotic 5-HT release by binding as a substrate to the 5-HT transporter (SERT) (Sitte and Freissmuth, 2010; Sáez-Briones and Hernández, 2013).

“Helping behaviour” refers to actions designed to assist an individual with a problem or to relieve her/his distress (Stukas and Clary, 2012; Chen et al., 2023) and is expressed by non-stereotyped complex responses that rely on both emotional and cognitive capabilities (Meyza and Knapska, 2018; Rigney and Hong, 2025). It is considered an empathy-based prosocial behaviour (Cronin, 2012), but the essential motivation for helping behaviour might still be a matter of debate (Vieira and Olsson, 2024). Nevertheless, it has been assumed that helping behaviour shares certain features with social interaction and can be considered an OXT-mediated behaviour, as knockout prairie voles for OXT receptors exhibit less capabilities to execute helping behaviour and less interest to help a victim congener (Kitano et al., 2022). Therefore, it may be hypothesised that MDMA should enhance this behaviour because of a combined 5-HT/OXT downstream mechanism. Nevertheless, studies about the effects of this prototypical entactogen on helping behaviour are lacking. Indeed, it has been reported that MDMA binding to SERT within the nucleus accumbens (NAc) is needed to induce prosocial behaviour (Heifets et al., 2019), but the contribution of the 5-HT released by MDMA in eliciting helping behaviour is still unknown. Besides, the development and persistence of prosocial behaviours require reinforcement through complex reward mechanisms, including long-term depression (LTD) linked to glutamatergic synaptic transmission coming from the prelimbic prefrontal cortex (prPFC) to the NAc (Dölen et al., 2013; Nardou et al., 2019). Additionally, studies claiming functional associations between neural plasticity undergoing mirror neurones of the anterior cingulate cortex (ACC) and the occurrence of action execution (Wu and Hong, 2022; Keysers and Gazzola, 2014) support the idea that helping behaviour depends on the activity of mirror neurones located in the ACC (Wu et al., 2022; Zhang M. et al., 2024). Therefore, the above data highlight the notion that combined neuroplasticity may also play a relevant role in driving and remembering prosocial decisions and actions related to MDMA-mediated effects.

In the present study, the evaluation of 5 sub-toxic dose levels (0.25 mg/kg, 0.5 mg/kg, 1 mg/kg, 5 mg/kg and 10 mg/kg i.p.) of MDMA on helping behaviour, has been conducted, using a behavioural setup based on the intrinsic aversion of rats to water. In addition, two complementary *in vivo* electrophysiological approaches were also conducted. Firstly, we investigated the acute effects of MDMA (10 mg/kg i.p.) on the induction of LTD in the NAc core of adult rats by stimulation of the dorsal raphe nucleus (DRN). Secondly, we studied the acute effects of MDMA (10 mg/kg i.p.) on the induction of long-term potentiation (LTP) evoked in the ACC of adult rats by electrical stimulation of the contralateral ACC. In addition, we also investigated whether the effects of MDMA on ACC LTP (if any) are mediated by 5-HT and OXT in light of reports that helping behaviour relies on OXT signalling in the ACC. This goal was achieved by micro-injecting the 5-HT_2A_ receptor antagonist ketanserin (Ketan) into the paraventricular nucleus (PVN) of the hypothalamus, where 5-HT has been described to regulate the activity of OXT neurones involved in social behaviours (Liu et al., 2023). Besides, the OXT receptor antagonist atosiban (Ato) was microinjected into the recorded ACC, where OXT could promote empathy-based behaviours (Zhang L. et al., 2024) downstream to its regulation by 5-HT.

## Materials and methods

### Animals

Male Sprague-Dawley rats (250–280 g in weight) were housed individually in a temperature-controlled vivarium (22 °C) for 10 days before starting the behavioural or the electrophysiological experiments. Animals were allowed to access food and water *ad libitum* and were kept under an inverted 12:12 day/night cycle. Individual rat checking was conducted daily before, during and after the experimental sessions. The experimental protocols and animal management followed the NIH Guide for the Care and Use of Laboratory Animals (National Research Council, 2011) and were approved by the Institutional Ethics Committee of the University of Santiago de Chile (protocol 084/2024). According to the approved protocol, each animal was evaluated daily both during acclimatisation and before, during, and after each experimental session in the following aspects: external appearance (10 criteria), spontaneous behaviour (27 criteria), and response to handling (11 criteria). Each criterion was assigned a different rating. On each occasion, a numerical value was determined that assessed their viability for use in the experiment, based on the evaluation of their emotional state. Not more of 2% of the animals were excluded from the experiments as a result of this evaluation.

### Drugs

MDMA was synthesised starting from the corresponding benzaldehyde by a base-catalysed Henry-Knoevenagel condensation and a substitution with nitroethane in ionic liquid (2-hydroxyethylammonium formate), followed by lithium aluminium hydride (LiAlH_4_) reduction. Briefly, 3,4-methylenedioxybenzaldehyde was converted to a β-nitrostyrene (Henry-Knoevenagel condensation with nitroethane) in an ionic liquid (HCOOH/NH_2_CH_2_CH_2_OH) (Bicak, 2005; Alizadeh et al., 2010). After purification by crystallisation, the reaction product was reduced to the amine with LiAlH_4_. Then, 1 g of the 4,5-methylenedioxyamphetamine (MDA, 5.6 mmol) obtained was dissolved in tetrahydrofurane (THF, 5 mL) and added dropwise with stirring to a solution of formic acetic anhydride (44.8 mmol) in THF (15 mL). The reaction was held at room temperature for 6 h. All volatile components were removed under vacuum, affording a red-coloured oil that was treated without purification with LiAlH_4_ (1 g) in THF (10 mL). The resultant free base (MDMA) was converted into a water-soluble hydrochloride salt by either dissolving the free base in ethyl ether and producing bubbling hydrochloric acid (HCl) gas or by mixing a solution of the base with 2-propanol and adding an exact equivalent of concentrated aqueous HCl. For pharmacological evaluation, MDMA (as hydrochloride) was freshly dissolved in saline (0.9% NaCl). The experimental protocol for the synthesis and use of MDMA were supervised and approved by the Institutional Ethics Committee of the University of Santiago de Chile (protocol 084/2024).

Atosiban (Ato) (Santa Cruz Biotechnology Inc., Dallas, TX, USA) was dissolved in saline. Ketanserin (Ketan) (Kayman Chemical, Ann Arbour, MI, USA) was dissolved in saline containing 5% dimethyl sulfoxide (DMSO).

### Apparatus to assess helping behaviour

**FIGURE 1:**
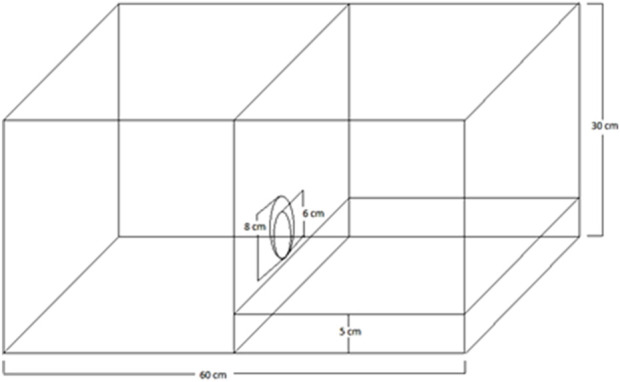
Schematic representation of the helping behaviour box, based on Sato et al. (2015), with modifications (see “helping behavioural evaluation”). A transparent acrylic wall separating the “dry” (right) and “wet” (left) compartments contains a pendular transparent door, allowing visual contact between rat pairs.

Behavioural experiments were conducted using a basic “helping behaviour box” as previously described (Sato et al., 2015) (Figure 1). It consists of an acrylic black box composed of 2 chambers of the same size (30 × 30 × 30 cm each): one chamber is a wet area, with the floor located 5 cm deep, forming a pool filled with 5 cm of water (“pool” chamber), while the other is a dry area (“dry” chamber). Chambers were separated by a transparent wall that featured a circular pendular door in the centre, which could be easily opened by sliding it to one side from the dry area. Fresh warm water was added manually and changed before each experiment using a drainpipe driven by gravity attached to the wall facing the pendular door.

### Helping behaviour evaluation

Evaluation of helping behaviour was conducted in a total of 67 rat pairs (134 rats), using an experimental approach based on a protocol already described (Sato et al., 2015), with modifications. Naïve animals were randomly assigned in pairs. In each rat pair, one rat was named “helper” and the other “soaked” (victim). The experimental protocols consisted of a sequence of 4 phases: (a) acclimatisation, (b) naïve animals’ cycle, (c) control tests and (d) role reversal rats’ cycle.

- Acclimatisation: all helper animals were individually placed in the “dry” chamber (Figure 1) for acclimatisation during 5 min on 3 consecutive days.
- Naïve animals’ cycle: starting at day 4, a cycle of 12 sessions (lasting a maximum of 5 min each) on consecutive days in naïve rat pairs was conducted. Helper rats were inoculated i.p. with saline solution (0.9% NaCl) or a specific MDMA-hydrochloride dose (0.25, 0.5, 1, 5 or 10 mg/kg) 30 min before each session. A typical experiment began when the helper rat and the soaked rat of a pair were placed simultaneously in the “dry” and in the “pool” chambers (Figure 1), respectively, and led to move freely. As daily sessions progressed, animals were randomly reassigned every day (i.e., each helper rat was paired with a different soaked rat every day) to prevent prior knowledge between both rats from being a motivating factor in helping behaviour performance until the end of the experiment at session 12. During each daily session, latency scores for door-opening (in seconds) and the number of door-openings (categorical binary “yes or no” counting) were measured by an investigator who was blinded to what was administered to the helper rat (MDMA or saline). In all cycles, only the first door-opening performed by the helper rat was counted as a successful helping behaviour event.
- Control tests: Three control tests were conducted following the conclusion of the naïve rats’ cycle: an “object” test, an “emptiness” test, and an “empathy” test. These tests were run only with helper rats that were successful in opening the door in session 12 of the naïve rats’ cycle. The “empathy” test was identical to a regular session, except that there was no soaked rat in the “pool” chamber. In the “empty” test, neither water nor the soaked rat was present. Finally, the “object” test was conducted with a dry “pool” chamber, and the soaked rat was replaced by a stuffed toy rat.
- Role reversal rats’ cycle: the day after completing the control tests, a new second 12-day session cycle was conducted using the same animals employed in the first 12-day session cycle, but with roles interchanged between pairs (role reversal); i.e., helper rats now became victims (soaked) and victims became helper rats. Again, as daily sessions progressed, each rat pair was randomly reassigned every day, meaning that each helper rat was paired with a different soaked rat every day. As for naïve animals, latency scores for door-opening (in seconds) and the number of door-openings (categorical binary “yes or no” counting) were measured during each daily session by an investigator who was blinded to what was administered to the helper rat (MDMA or saline). In all cycles, only the first door-opening performed by the helper rat was counted as a successful helping behaviour event.

### Electrophysiological evaluation

For all electrophysiological experiments, a total of 20 male Sprague-Dawley rats were used. Animals were anaesthetised with 1.5 g/kg urethane, tracheostomised and placed in a stereotaxic apparatus. Adequate ventilation was kept during the experiments using a respirator pump. Animals never regained consciousness, and no changes in heart rate in response to stimulation were detected throughout the experiments.

1-mm diameter holes drilled in the frontal bone allowed for the positioning of both the stimulating electrode (two side-by-side glued 50-µm diameter insulated tungsten wires with a 0.5-mm tip separation) and the recording electrode (a 20-µm tip diameter insulated tungsten semi-microelectrode, regarding another electrode located on the excised muscles over the frontal bone) into the selected brain regions. Stereotaxic coordinates for electrode positioning were taken from the atlas of the rat brain by Paxinos and Watson (2007). Electrical stimuli were rectangular-wave pulses produced by a Grass S11 stimulator, which was equipped with a Grass SIU-5 stimulus isolation unit and a Grass CCU 1A constant current unit (all Grass equipment from Astro-Med Inc., West Warwick, RI, United States). The evoked field potentials (EFPs) were amplified using a Grass P-511 preamplifier (0.8–300 Hz bandwidth), digitised, averaged, and presented on a Tektronix TBS 1052C digital oscilloscope (Beaverton, OR, United States)). Baseline EFPs recordings were obtained after a stabilisation period lasting up to 30 min, followed by the administration of either 10 mg/kg MDMA or saline injected i.p. 30 min before the application of an LTP or LTD induction protocol. Positive (P) and negative (N) peak latencies and the P-N peak-to-peak amplitude of averaged EFPs were measured using time and voltage cursors provided by a digital oscilloscope. Time-course curves were obtained by measuring the peak-to-peak amplitude changes induced by MDMA or saline before and after applying the synaptic plasticity induction protocol. Areas under the curves were calculated as the integral from 0 to 60 min following the application of the protocol.

### Recording and assessment of LTD in the NAc core

EFPs were elicited in the right NAc core (stereotaxic coordinates A: 2.5, L: 1.5 mm from the bregma, V: - 5.5 mm from the cortical surface) by electrical stimulation of the ipsilateral prelimbic prefrontal cortex (prPFC, A: 3.0, L: 0.8 mm from bregma; V: - 2.8 mm from the cortical surface), as already described (Morgan et al., 2022). Test EFPs were elicited by stimulating the right prPFC with single rectangular electrical pulses of 0.2 ms duration and 0.2 Hz frequency, with just the current intensity required to evoke 500 µV peak-to-peak EFPs in the NAc core. Long-term depression (LTD) was elicited by stimulating the right side of the dorsal raphe nucleus (DRN, coordinates A: - 7.9, L: 0.2 mm from bregma; V: - 5.7 mm from the brain surface) with a low-frequency train of 900 rectangular electrical pulses (0.2 ms, 5 Hz, 1.5 x test current intensity) using two side-by-side glued 50-µm diameter insulated tungsten wires with a 0.5-mm tip separation, a protocol that would elicit 5-HT-mediated LTD of EFPs in the NAc core. MDMA (10 mg/kg i.p.) was administered 30 min before the application of the 900-pulse protocol. To assess the LTD magnitude, decreases (in percentage) of the peak-to-peak amplitude of EFPs were plotted as time-course curves, and the areas under the curves were calculated.

### Recording and assessment of LTP in the ACC

EFPs were elicited in the right ACC (stereotaxic coordinates A: 2.7, L: 0.8 mm from bregma; V: - 1.6 mm from the cortical surface) by electrical stimulation of the contralateral ACC (same coordinates, but in the left hemisphere). Test EFPs were elicited by stimulating the left ACC with single rectangular electrical pulses (0.2 ms duration, 0.2 Hz frequency), with a current intensity enough to evoke 500 µV peak-to-peak EFPs in the contralateral ACC. Transcallosal LTP-like plasticity was elicited by applying a high-frequency stimulation protocol that elicits postsynaptic LTP in ACC pyramidal neurones previously reported (Sah and Nicoll, 1991) and modified for *in vivo* experiments (Sáez-Briones et al., 2023). It consisted of two consecutive pulse trains (100 pulses of 0.2 ms duration each, at 100 Hz frequency) with a 20 s inter-train interval and with 2x test current intensity. To assess the potential role of both 5-HT and OXT in the effect of MDMA, 5 µg of either the 5-HT_2A_ receptor antagonist Ketan (dissolved in 0.5 µL of saline containing 5% DMSO) or the OXT receptor antagonist Ato (dissolved in 0.5 µL of saline) were respectively microinjected into the right PVN or the right ACC over a 45-s period using a Hamilton syringe mounted in a micromanipulator. This was done 5 min before the insertion of the recording semi-microelectrode, immediately followed by an i.p. injection of 10 mg/kg of MDMA. To assess the LTP magnitude, changes (in percentage) of the peak-to-peak amplitude increase of EFPs were plotted as time-course curves, and the areas under the curves were calculated.

### Statistical analysis

All statistical analyses were performed using Prism software version 8.1.0 (GraphPad, San Diego, CA, United States). Behavioural data are presented as mean ± SEM, including the binary categorical “yes or no” data related to the number of door-openings, which are presented as the percentage of rats in a group that open the door in each daily session. The effects, both on the latency of door-opening and on the percentage of rats in a group that open the door throughout the daily helping behaviour sessions, were analysed using a two-way repeated measures ANOVA. The temporal evolution of the effects in the control rats was analysed using linear regression. For both control rats and those injected with MDMA, the comparison of the effects on door-opening latency and the percentage of door-openings was performed through the calculation of the areas under the curves over the sessions, followed by an unpaired two-tailed Student's t-test or a one-way ANOVA associated with the Holm-Sidak *post hoc* test. Electrophysiological data are presented as mean ± SEM. The induction and maintenance of LTP or LTD were analysed using a two-way ANOVA, followed by a Holm-Sidak *post hoc* test for multiple comparisons (if applicable). The effects of MDMA were measured by comparing the areas under the curves of two or more test groups. Data were analysed using an unpaired two-tailed Student's t-test or a one-way ANOVA followed by a Holm-Sidak *post hoc* test. In all experiments, a probability level of 0.05 or lower was accepted as significant.

## Results

### Behavioural evaluation

**FIGURE 2:**
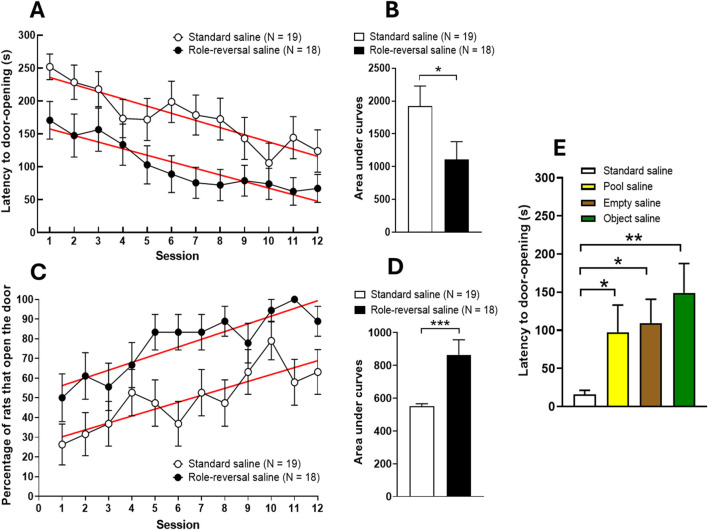
Latencies to door-opening and percentages of door-opening during the naïve cycle of helping behaviour and comparison of latency scores in “empathy”, “emptiness”, and “object” control experiments. Values are presented as means ± SEM of latencies to door-opening **(A)**, means ± SEM of the percentage of rats that open the door **(C)** and as areas under the curves [**(B)** and **(D)**, respectively]. Regression lines are red-coloured. For control experiments **(E)**, only latency scores from helper rats that opened the door at session 12 before the fixed maximum 5-min cut-off limit (N = 13) were considered. Colored bars correspond to scores from helper rats submitted to “empathy” (N = 9), “emptiness” (N = 8), and “object” (N = 5) experiments, respectively. Values are presented as means ± SEM of latencies to door-opening. Each helper rat was inoculated with saline (1 mL/kg i.p.) 30 min before starting the experiment.

As shown in Figure 2A, two-way repeated measures ANOVA detected significant differences in latency scores as sessions progressed (*P* ANOVA session <0.0001, *F* (11,385) = 7.067) and between the two protocols (*P* ANOVA protocol = 0.0147, F (1,35) = 6.593) for naïve animals. No significant interaction was detected between the two factors (*P* ANOVA session × protocol interaction = 0.7614, F (11,385) = 0.6761). Besides, the difference in the slopes is not significant (F test, *p* = 0.6571, F (1,20) = 0.2031), meaning that latency scores decreased similarly across sessions in both rat pair groups. Analysis of the areas under the curves of the latency data from rats subjected to role reversal (helper rats that had formerly been soaked rats) showed that these animals overall displayed significantly lower scores for latency to door-opening across sessions as compared to naïve helper rats before role reversal (unpaired Student’s t-test, *p* = 0.0453; t = 1,983, df = 35, Figure 2B). On the other hand, two-way repeated measures ANOVA showed significant differences in the number of door-openings as sessions progressed (*P* ANOVA session <0.0001, F (11,385) = 6.173) and between the two protocols (*P* ANOVA protocol = 0.0063, F (1,35) = 8.452). No significant interaction was detected between the two factors (*P* ANOVA session × protocol interaction = 0.4744, F (11,385) = 0.9688, Figure 2C). In addition, the percentage of helper animals that opened the door increased steadily over the course of sessions in both naïve helper rats and those that were subjected to role reversal. The increases in door-opening were similar across sessions, as evidenced by the non-significant difference in slopes of regression lines between the two groups of rats (F test, *p* = 0.6571, F (1,20) = 0.2031, Figure 2C). Analysis of the areas under the curves indicated that the percentage of helper animals that opened the door was significantly higher in the group that was subjected to the role reversal protocol than in the naïve group (unpaired Student’s t-test, *p* = 0.0016; t = 3,429, df = 35, Figure 2D). Conversely, as shown by one-way ANOVA (*P* = 0.0284, F (3,44) = 3.315), followed by the Holm-Sidak’s multiple comparisons *post hoc* test (Figure 2E), control experiments showed that door-opening latencies were significantly increased after removing the soaked rat from the pool chamber (“empathy” test) (*p* = 0.0436), the water and the soaked rat from the pool chamber (“emptiness” test) (*p* = 0.0436), and the water from the pool chamber and replacing the soaked rat with a stuffed rat (“object” test)(*p* = 0.0090).

**FIGURE 3:**
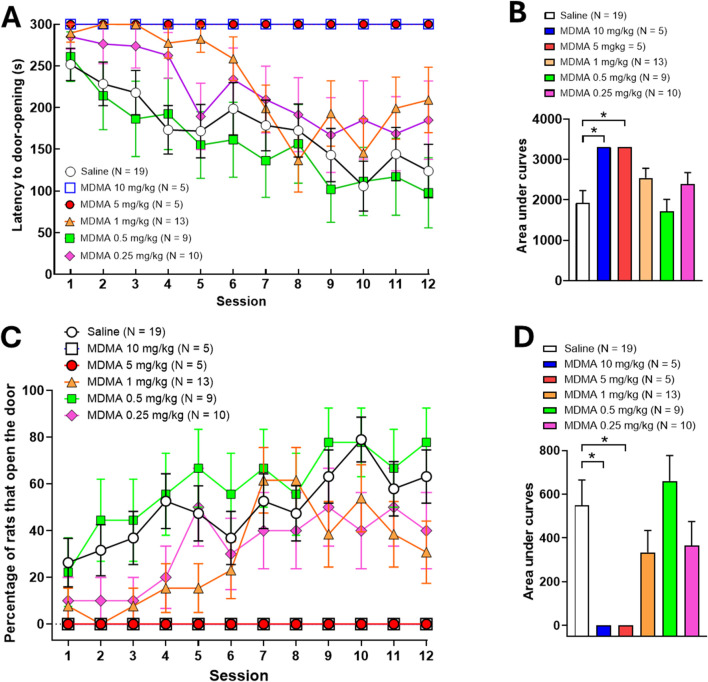
Effects of MDMA (10, 5, 1, 0.5 or 0.25 mg/kg i.p.) in the latency to door-opening and the percentage of rats that open the door over the course of daily sessions in rat pairs following the naïve protocol in the helping behaviour paradigm. Values are presented as means ± SEM of latencies to door-opening **(A)**, means ± SEM of the percentage of rats that open the door **(C)** and as areas under the curves [**(B)** and **(D)**, respectively]. Each animal acting as a helper rat was inoculated with MDMA (i.p.) 30 min before starting the experiment.

As shown in Figure 3A, two-way repeated measures ANOVA indicated significant differences in latency scores as sessions progressed (*P* ANOVA session <0.0001, F (11,605) = 6.534) and among different MDMA doses (*P* ANOVA dose = 0.0020, F (5,55) = 4.362). No significant interaction was detected between the two factors (*P* ANOVA session × dose interaction = 0.3487, F (55,605) = 1.068). Scores of the areas under the curves analysed with one-way ANOVA yielded significant differences (*P* ANOVA = 0.0105, F (5,55) = 3.341) in latency scores between MDMA-treated helper rats compared to control helper rats. The Holm-Sidak’s *post hoc* multiple comparison test applied to the corresponding areas under the curves showed a significant detrimental effect for the two higher MDMA doses (10 mg/kg and 5 mg/kg) evaluated on the latency to door-opening by naïve helping rats, as the latency to door-opening was increased over the cut-off limit of 5 min (*p < 0.05) for these two MDMA doses, resulting in no door-opening. In contrast, the three lower MDMA doses (1 mg/kg, 0.5 mg/kg and 0.25 mg/kg) were indistinguishable from saline (Figure 3B). A similar detrimental trend was observed in the number of door-openings after MDMA administration: Two-way repeated measures ANOVA detected significant differences in door-opening scores as sessions progressed (*P* ANOVA session <0.0001, F (11,605) = 4.542) and among different MDMA doses (*P* ANOVA dose = 0.0006, F (5,55) = 5.109). Again, no significant interaction was detected between the two factors (*P* ANOVA session × dose interaction = 0.5406, F (55,605) = 0.9690, Figure 3C). On the other hand, one-way ANOVA detected significant differences in scores among treatments (*P* ANOVA = 0.0066, F (5,55) = 3.629). Subsequent Holm-Sidak’s *post hoc* multiple comparison test for the corresponding areas under the curves indicated that doses of 10 and 5 mg/kg of MDMA suppressed door-opening behaviour in naïve helping rats (*p* = 0.0318), while for 1 mg/kg, 0.5 mg/kg and 0.25 mg/kg of MDMA, the percentage of rats that opened the door was not significantly different from that of helper rats receiving saline (Figure 3D).

**FIGURE 4:**
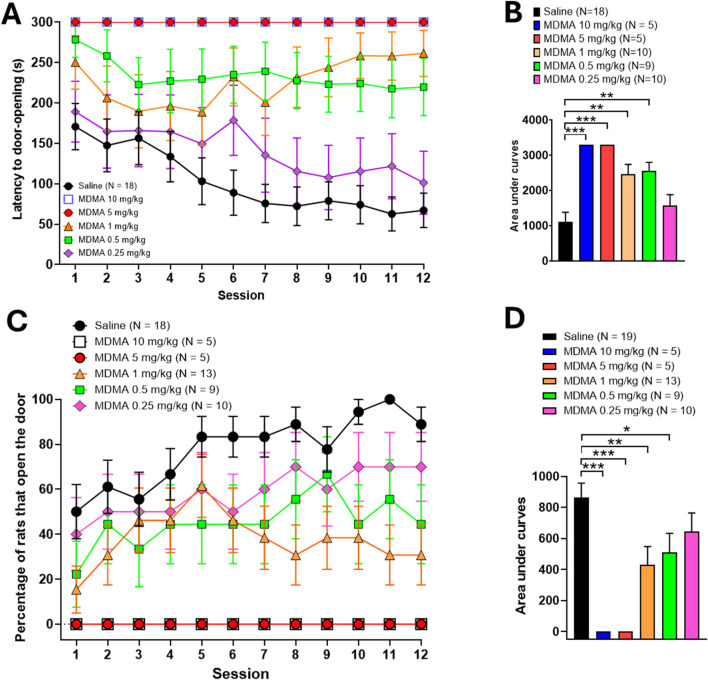
Effects of MDMA (10, 5, 1, 0.5 or 0.25 mg/kg i.p.) in the latency to door-opening and the percentage of helper rats that open the door over the course of daily sessions in rat pairs following the role-reversal protocol in the helping behaviour paradigm. Values are presented as means ± SEM of latencies to door-opening **(A)**, means ± SEM of the percentage of rats that open the door **(C)** and as areas under the curves [**(B)** and **(D)**, respectively]. Each animal acting as a helper rat was inoculated with MDMA (i.p.) 30 min before starting the experiment.

Figure 4 shows for the role-reversal group that two-way repeated measures ANOVA detected significant differences in latency scores as sessions progressed (*P* ANOVA session = 0.0471, F (11,561) = 1.824) as well as in scores among different treatments (*P* ANOVA treatment <0.0001, F (5,51) = 7.565). Besides, a significant interaction was detected between the two factors (*P* ANOVA session × dose interaction = 0.0042, F (55,561) = 1.621, Figure 4A). The areas under the curves analysed with one-way ANOVA yielded significant differences (P ANOVA = 0.0001, F (5,54) = 8,687) in latency scores between MDMA-treated helper rats compared to control helper rats. Subsequent *post hoc* analysis with the Holm-Sidak’s multiple comparisons test showed a significant detrimental effect for the four higher MDMA doses (10 mg/kg and 5 mg/kg, p < 0.001; 1 mg/kg and 0.5 mg/kg, p < 0.01) for latency scores, while the lower MDMA dose (0.25 mg/kg) was indistinguishable from saline (Figure 4B).

A similar adverse trend was observed in the number of door-openings after MDMA administration to role-reversal helper rats: Two-way repeated measures ANOVA detected significant differences in door-opening scores as sessions progressed (*P* ANOVA session <0.0127, F (11,594) = 2.210) and among different MDMA doses (*P* ANOVA dose = 0.0001, F (5,54) = 7.667). No significant interaction was detected between the two factors (*P* ANOVA session × dose interaction = 0.4546, F (55,594) = 1.012, Figure 4C). Analysis of the corresponding areas under the curves by means of one-way ANOVA detected significant differences in scores among treatments (*P* ANOVA <0.0001, F (5,54) = 7.730). Subsequent *post hoc* analysis with the Holm-Sidak’s multiple comparisons test yielded significant differences (*p < 0.05, *p* < 0.01, *p < 0.001) in scores of the corresponding areas under the curves from role-reversal MDMA-treated helper rats compared to role-reversal control helper rats receiving saline (Figure 4D).

### Electrophysiological evaluation

**FIGURE 5:**
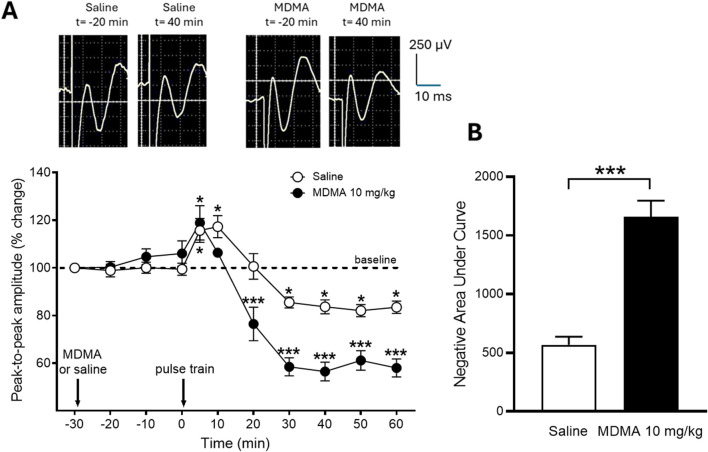
Effect of MDMA (10 mg/kg i.p.) on LTD evoked in the NAc core as assessed by *in vivo* electrophysiology. **(A)**: Time-courses of the EFPs elicited in the NAc core by stimulation of the ipsilateral prPFC expressed as changes (%) in peak-to-peak amplitude of EFPs after saline or MDMA injection followed by application of the LTD-inducing protocol, with respect to the pre-saline/MDMA control. Values are presented as means ± SEM (N = 4; 12 responses were averaged per animal for each data point). Representative examples of the average of 12 successive EFPs elicited in the NAc core by stimulation of the ipsilateral prPFC from two animals (one injected with saline and the other with MDMA) recorded at minute −20 and minute −40 (see calibration bars) are shown above the graph. **(B)**: Effects of saline and MDMA on NAc core LTD expressed as areas under the corresponding time-course curves. (*) indicates statistical significance (**p < 0.01, ***p < 0.001, ****p < 0.0001) according to Holm-Sidak multiple comparison *post hoc* test.

The neuroplastic effects of MDMA (10 mg/kg) were assessed in adult rats by inducing LTD in the NAc core via a low-frequency protocol (900 pulses) applied to the DRN and by inducing LTP in the ACC through a high-frequency stimulation protocol applied to the contralateral ACC (Figure 5). The low-frequency protocol in the DRN induced a transient phase of substantial EFP potentiation (*p* < 0.05), subsequently succeeded by a notable LTD of EFPs. This effect was evidenced by a sustained reduction in the peak-to-peak amplitude of EFPs induced in the NAc core from the prPFC, as revealed by two-way ANOVA (*P* ANOVA time <0.0001, F (10,66) = 41.89) followed by the Holm-Sidak´s multiple comparisons *post hoc* test (*p* < 0.05, Figure 5A). The administration of MDMA did not alter the brief duration of EFP potentiation. Instead, it resulted in a substantial enhancement of the LTD initiated in the NAc core (i.e., EFPs exhibited a more pronounced depression in peak-to-peak amplitude) when contrasted with the LTD observed in saline-treated animals (***p < 0.001). This effect continued throughout the recording period, as revealed by the corresponding analysis of the areas under the curves (***p < 0.001, t = 7.126, df = 6; unpaired Student's *t*-test, Figure 5B).

**FIGURE 6:**
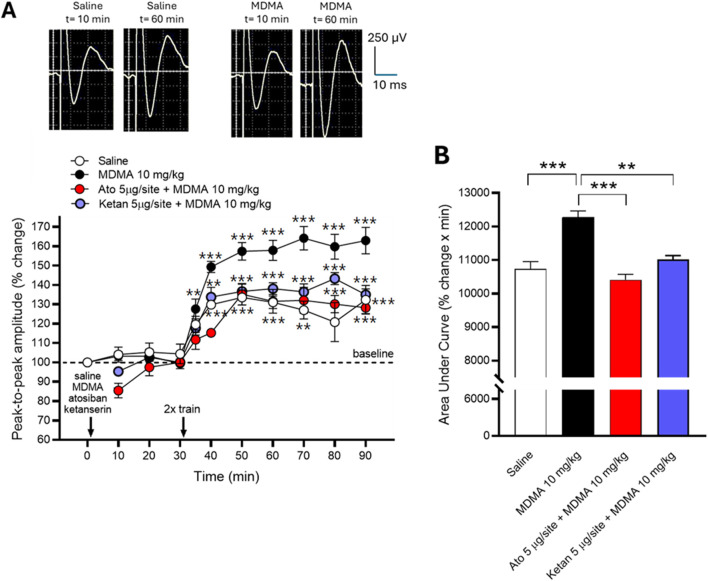
Effects of MDMA (10 mg/kg i.p.), OXT antagonist Ato and 5-HT_2A_ antagonist Ketan on LTP evoked in the ACC as assessed by *in vivo* electrophysiology. **(A)**: Time-courses on transcallosal EFPs elicited in the right ACC by stimulation of the contralateral one, the effect of microinjecting Ato (5 µg/site) into the recording site (ACC) and Ketan (5 µg/site) into the ipsilateral PVN. Values are expressed as changes (%) in peak-to-peak amplitude of transcallosal EFPs followed by application of the LTP-inducing protocol, with respect to the pre-saline/MDMA control and presented as means ± SEM (N = 4; 12 responses were averaged per animal for each data point). Representative examples of the average of 12 successive EFPs elicited in the ACC by stimulation of the contralateral ACC from two animals (one injected with saline and the other with MDMA) recorded at minute −10 and minute −60 (see calibration bars) are shown above the graph. **(B)**: Effects of saline, MDMA (10 mg/kg i.p.), Ato 5 µg + MDMA, or Ketan 5 µg + MDMA on ACC LTP expressed as areas under the corresponding curves. (*) indicates statistical significance (**p < 0.01, ***p < 0.001) according to Holm-Sidak multiple comparisons *post hoc* test.

As shown in Figure 6, two-way ANOVA showed that application of the high-frequency stimulation regimen to the left ACC resulted in LTP induction in the right ACC, as revealed by significant increases in the peak-to-peak amplitude of the EFPs (*P* ANOVA time <0.0001, F (10,110) = 69.82; *P* ANOVA treatment <3.110, F (3,110) = 42.77; *P* ANOVA time × treatment interaction <0.0001, F (30,110) = 3.072). Holm-Sidak’s multiple comparisons *post hoc* test was used to compare all values obtained after saline or MDMA against the pre-saline/MDMA respective control value recorded at minute zero, except for the Ato + MDMA and the Ketan + MDMA groups, where values obtained after the 2x train stimulation were compared to the last pre-train value recorded at minute −30 (**p < 0.01, ***p < 0.001, Figure 6A). Prior microinjection of the 5-HT_2A_ receptor antagonist Ketan (5 µg) into the ipsilateral PVN or the OXT receptor antagonist Ato (5 µg) into the recorded ACC fully inhibited the effects of MDMA, as revealed by one-way ANOVA (*P* ANOVA = 0.0001, F (3,10) = 20.11) followed by Holm-Sidak’s multiple comparisons *post hoc* test for the corresponding areas under the curves (**p < 0.01, p < 0.001, Figure 6B).

## Discussion

The aim of the present work was to evaluate the effects of sub-toxic doses of the synthetic entactogen MDMA on helping behaviour in male adult rats using an already described experimental setup based on the intrinsic aversion of these animals to water, with some modifications. The results obtained using the modified protocol (Figures 2A–C) are consistent with naïve control data already published, including the control tests (“empathy”, “emptiness”, and “object”), ensuring that the helper rat’s performance seems to occur only when there is a real rat to help (Figure 2E) (Sato et al., 2015). Indeed, the shorter latency in opening the door to rescue the soaked rat from the water trap results from the helper rats’ motivation, which may be triggered by sensory signals (olfactory, acoustic, and visual) emitted by the soaked rat and absent in the control groups, as has already been proposed in other studies (Arakawa, 2021). Nevertheless, one open question is to find out whether the partial maintenance of the “go” reaction (the latency to open the door increased in the control tests, but the helper rats still opened the door) in response to a “no-go” signal (absence of the soaked rat) is due to a learnt component, operating beyond the motivational component determined by social sensory cues. This may suggest that a learnt aspect, involving some form of long-term memory, influences helpful behaviour as sessions progress. Similar behavioural results are frequently observed in memory testing of learnt behaviours over multiple sessions, such as in the 8-arm radial maze test for spatial learning and memory (Soto-Moyano et al., 2005), the Morris water maze (Wenk, 2004), and during operant conditioning in a Skinner box (Burgos et al., 2010). Indeed, performance improvements across trials and phases that resemble learning curves have already been reported using the present (Sato et al., 2015) or other helping behaviour paradigms (e.g., see Silva et al., 2020).

The behavioural data obtained for MDMA showed that the drug disrupts helping behaviour along the subtoxic dose range evaluated. Although the scores of latencies to door-opening and the percentage of door-openings achieved by MDMA-treated helper rats are not strictly correlated when running the standard paradigm, they appear to correlate after role reversal. Full inhibition of door-opening by the two higher doses of MDMA was verified even after role reversal, thus highlighting the fact that previous experience did not compensate for the inhibitory effect elicited by MDMA. Besides, lower doses (1 mg/kg and 0.5 mg/kg) exerted partial inhibition only after role reversal, whereas the lowest dose (0.25 mg/kg) was fully inactive. These data support the notion that the effects elicited by MDMA may reflect an inhibitory mechanism that is expressed differently at high and low subtoxic doses. In this regard, the results obtained might support the notion that the occurrence of helping behaviour results from the association of an emotional and a cognitive (learnt) component, both probably inhibited by the 5-HT released by MDMA at high doses. In contrast, lower doses might inhibit only the cognitive component, as dose-dependent behavioural inhibition was observed only after role reversal. These results do not agree with the assumption that MDMA should enhance prosocial behaviour. As already known, the enhancement of adjacent-lying behaviour in the classical social interaction paradigm has become a pharmacological hallmark of MDMA in the subtoxic dose range after acute administration, which can be modulated specifically (Sáez-Briones et al., 2019). Further, MDMA may not only promote prosocial behaviour but also diminish or even abolish aggression (for a review, see Kamilar-Britt and Bedi, 2015). Importantly, adjacent lying enhancement occurs regardless of hyperlocomotion (see below), an always typical psychomotor effect of MDMA at subtoxic doses, highlighting the fact that prosocial behaviour is not disrupted by motor impairment (McGregor et al., 2008). Beyond this evidence, MDMA seems to even further act as an empathy enhancer, as recently demonstrated for the first time in the social transfer of pain and analgesia paradigm (Rein et al., 2024). Nevertheless, prosocial behaviour enhancement seems to be conditioned by several factors (e.g., age, dose administration regimen, housing conditions, gender) that may lead to disruptive effects after administration of MDMA. For instance, it has been shown that pretreatment with high doses of MDMA may reduce social interaction after a withdrawal period in adolescent (Fone et al., 2002), or adult rats (Thompson et al., 2008). Moreover, MDMA in doses up to 1 mg/kg failed to produce reinforcement effects in male and female pair-housed rats (Smith et al., 2021). Besides, a very recent publication reported that acute administration of MDMA (5 mg/kg) in young male but not female adult rats, irrespective of the housing conditions, reduce social behaviour, as measured in the social preference test (Palacios-Lagunas et al., 2026). Given that helping behaviour is defined as a specific form of prosocial behaviour, and although some results from these investigations stem from acute instead of chronic MDMA administration in social interaction models, the current findings partially align with this previous evidence.

Notwithstanding the above, the disruptive effect of MDMA at high doses on helping behaviour could be related to the effects on the baseline anxiety levels of rodents reported for the drug. Among its multiple potential therapeutic applications, MDMA has been proposed as a potentially effective drug for the treatment of social anxiety disorders and the post-traumatic stress disorder in humans (Heifets and Malenka, 2016). In rodents, MDMA exhibits a mixed profile over anxiety, as measured in the elevated plus maze: whereas low acute and subchronic subtoxic doses have been described to produce anxiogenic effects, high doses produce anxiolytic effects (Lin et al., 1999). Interestingly, this profile seems to arise at acute but not after chronic treatment with MDMA (Ho et al., 2004). Other works do not agree with this finding, indicating that the anxiogenic-like effects observed after acute administration of MDMA not only persist but also are increased after sub chronic treatment (Navarro and Maldonado, 2002). Regardless of the differences in doses levels between these results and those obtained previously by us showing that MDMA (10 mg/kg) is anxiolytic and MDMA (5 and 1 mg/kg) have no effect on basal anxiety in the elevated plus maze (Quinteros-Muñoz et al., 2010), it would be relevant to verify if MDMA is capable of emulating the response profile of anxiolytic drugs that produce a decrease in helping behaviour in rats associated with the preference for a treat (Ben-Ami-Bartal et al., 2016). If this were the case, one may propose that the link between dose level and the occurrence of MDMA-mediated anxiolytic/anxiogenic-like effects might explain both the inhibition of helping behaviour observed at 10 and 5 mg/kg and the partial inhibition observed at 1 mg/kg after role reversal. Further research is needed to explore this possibility.

Despite the latter, it is reasonable to be cautious in the interpretation the disrupting effects on helping behaviour observed by MDMA, as some confounding factors associated with the drug itself and the protocol conditions might be relevant to consider. In this context, one distinctive spontaneous psychomotor effect at high doses of MDMA (5–10 mg/kg) in rodents is hyperlocomotion, which is rather exclusive of grooming and rearing behaviours after acute administration (Quinteros-Muñoz et al., 2010). Although current evidence is controversial regarding the influence of hyperlocomotion on prosocial behaviours (e.g., social interaction, Šlamberová et al., 2015), several studies have been published showing that motor hyperactivity induced by drugs structurally related to MDMA (e.g., D-amphetamine) does not affect prosocial behaviour (Sams-Dodd, 1995; Sams-Dodd, 1996; Sams-Dodd, 1998). Notwithstanding the above, it should be noted that the disruptive effects reported for MDMA in the present work not only occur at doses where hyperlocomotion has been reported, but also partial inhibitory effects are observed at 1 mg/kg and 0.5 mg/kg, which correspond to doses where hyperlocomotion does not occur (Quinteros-Muñoz et al., 2010). Besides, in other behavioural protocols designed to evaluate cognitive performance (e.g., the Olton radial maze), it has been reported that low doses of MDMA produce disruptive effects on learning without affecting locomotion, which points to a dissociation between the disruptive effect of MDMA on learning and its effects on motor activity (Braida et al., 2002). Besides, it is also important to mention that no evidence of hyperlocomotion was found in the helper rats within the apparatus used during the helping behaviour experiments, nor in naive rats inoculated with MDMA, nor after role reversal. This finding is notable, as it contrasts with the known results for MDMA in classic psychomotor activity assessment experiments (Páleníček et al., 2007; Quinteros-Muñoz et al., 2010). One may speculate that this discrepancy might be related to a context effect associated with the experimental box itself or the presence of the soaked rat in the perceptual environment of the helper rat.

On the other hand, behavioural alterations (psychomotor or emotional) induced after chronic administration of MDMA in rodents are described to occur as a result of behavioural sensitization. Achieving sensitization requires a protocol involving an aggressive administration regimen that may imply much higher total doses than those evaluated in the present work (Ball et al., 2009; Plocinski and Ball, 2022; Modi et al., 2006; Kalivas et al., 1998), a specific withdrawal or deprivation period needed to produce it (Magendzo and Bustos, 2003; Rothwell et al., 2010), and a defined context (Ball et al., 2006). In this regard, it should be noted that the administration regimen of the doses evaluated in the present work, besides being in the subtoxic range, should not meet the requirements to assume the induction of sensitization. However, it should also be noted that even in the absence of a deprivation period, there is previously published evidence showing the presence of locomotor sensitization for administration regimens similar to the one used in the present work (Ball and Slane, 2014; Aberg et al., 2007). Consequently, the possibility of generating some degree of sensitization associated with the administration regimen used in the present work should not be ruled out.

With regard to the protocol conditions used, it should be considered that it has been reported that prior acquaintance between animals increases helping behaviour (Ben-Ami Bartal et al., 2014). Therefore, in the present work animals were housed in individual cages to prevent prior acquaintance among animals from influencing the evaluation of the effects of MDMA on helping behaviour. Certainly, as social isolation may affect the basal emotional state of the animals (Mumtaz et al., 2018), this factor might act as confounding in data interpretation. Nevertheless, as previously discussed, animals injected with saline under the same isolation conditions of those treated with MDMA, successfully induced helping behaviour (see Figure 2). These results support the notion that the isolation period included in the modified protocol used in the present work do not affect the occurrence of helping behaviour. Besides, baseline behavioural alterations in rodents associated with social isolation occur more in adolescent than in adult rats (Rivera-Irizarry et al., 2020). Further, the acclimatisation time for the animals in the present work falls within the reported range of isolation that does not produce depressive or anxiogenic effects, and also promotes social interaction (Csikós et al., 2024). In addition, although it has been reported that social isolation does not promote social sensitization, isolated animals seem to exhibit the same social interaction capabilities compared to non-sensitized congeners (Curry et al., 2019). Nevertheless, it has been reported that under a much milder dose administration regimen that those usually associated with sensitization, the administration of MDMA (5 mg/kg) induces motor sensitization. Interestingly, these effects were shown to be higher in socially housed animals compared to isolated animals (Starosciak et al., 2012).

The *in vivo* electrophysiological data obtained following the administration of MDMA (10 mg/kg) showed a significant reinforcement of LTD elicited from the DRN in the NAc core. Additionally, a significant increase in transcallosal-evoked, 5-HT_2A_/OXT-dependent LTP was observed in the ACC. Despite this, the neuroplastic effects elicited by the drug appear to align with those mechanisms that promote prosocial/empathic behaviour (Ruzal et al., 2026). It is important to mention that all experiments were conducted *in vivo* in anaesthetised rats using urethane, which ensures anaesthesia quality while maintaining physiological conditions close to the awake state. In this regard, it should be considered that rats under urethane anaesthesia may show LTPs of lesser magnitude compared to non-anaesthetised controls. However, these effects can be compensated by increasing the tetanic stimuli, indicating that urethane anaesthesia preserves neuroplastic processes (Riedel et al., 1994). In addition, urethane is the preferred long-lasting anaesthetic in rodent electrophysiology when survival is not required, as it produces stable brain states akin to sleep (non-REM/REM), causes minimal cardiorespiratory depression, and provides excellent analgesia and muscle relaxation. Therefore, recordings, and data acquisition are stable and suitable for long-term experiments (Silver et al., 2021). These conditions enable also stable LTD recordings, as observed in the present work.

The neuroplastic effect of MDMA on LTD in the NAc core, described here *in vivo* for the first time, has been previously shown *in vitro* using voltage clamp in neurones from slices containing the NAc. This demonstrated that MDMA mimicked the actions of 5-HT in its ability to induce LTD (Heifets et al., 2019). Mechanistically, the enhanced LTD of EFPs in the NAc core induced by MDMA that we describe here could be reflecting a previously reported 5-HT_1B_ receptor-mediated, 5-HT-dependent presynaptic LTD that would inhibit glutamate release in axon terminals connecting the prPFC to the NAc core (Dölen et al., 2013; Nardou et al., 2019). This view is supported by the fact that 5-HT_1B_ receptors function as autoreceptors and heteroreceptors in axon terminals, regulating the release of various neurotransmitters, including 5-HT itself (Sari, 2004). This form of LTD has been positively correlated with the prosocial effects of MDMA (Nardou et al., 2019; Heifets et al., 2019). On the other hand, the role of OXT in MDMA-triggered NAc LTD and NAc-dependent social reward has given rise to rather conflicting results. Indeed, OXT-dependent 5-HT_1B_ receptor-mediated reinstatement of both NAc LTD and social reward after MDMA administration has been reported in adult mice (Nardou et al., 2019). In contrast, other studies found no influence of OXT receptor antagonists or genetic deletion of the receptor on MDMA-induced 5-HT_1B_ receptor-dependent LTD in the NAc and the associated prosocial effect in mice (Heifets et al., 2019). Despite the disagreement, both studies concur that both the prosocial effect and the 5-HT-dependent LTD in the NAc induced by MDMA require 5-HT_1B_ receptor activation. It should be noted that the LTD we observed in the NAc core is not only instrumental for the prosocial effects of MDMA but probably also for the motivational drive for helping behaviour. In fact, helping behaviour requires consecutive sequential processes, including a motivational drive (i.e., the eagerness to help), followed by a process comprising cognitive control and motor execution of helping operant actions that can be learnt through repetition (Rigney and Hong, 2025). In this regard, the NAc is a key hub involved in social approach and motivation. Here, glutamatergic inputs from various cortical regions (e.g., the ACC), conveying contextual, affective, and executive information relevant to social behaviour, were integrated. In this way, the motivational value of social stimuli can be shaped by regulating OXT and 5-HT via an LTD-based mechanism, allowing for fine-tuning of social reward (Dölen et al., 2013; Murugan et al., 2017). Based on this, the enhanced LTD process observed in the NAc core after MDMA administration could likely be related to an enhanced motivational drive for helping behaviour, yet it does not appear to be connected to the disrupted execution of helping operant actions observed during behavioural testing of MDMA effects, which likely depends on output brain regions that facilitate the execution of helping behaviour downstream from the NAc (i.e., the basal ganglia). The NAc integrates motivational and reward-related information with motor planning, sending signals that influence downstream motor output through its projections to the ventral pallidum and other basal ganglia structures. These downstream areas then influence brainstem centres that control movement, helping translate motivation into action (Grillner and Robertson, 2015; Morrison et al., 2017).

On the other hand, the increased transcallosal LTP in the ACC induced by MDMA, is likely due to the non-exocytotic release of 5-HT in several target regions of the brain. Agreeingly, it has been reported that activation of 5-HT_1A_ receptors increases LTP in the mouse prefrontal cortex, although the pre- or postsynaptic nature of the LTP was not approached (Meunier et al., 2013). However, ionophoretically applied 5-HT has been found to inhibit pyramidal cell firing in the prefrontal cortex (Rueter et al., 2000). Therefore, it is difficult to reconcile the enhanced LTP we found in the ACC with a direct neuronal effect of 5-HT. In fact, the LTP enhancement caused by MDMA-released 5-HT in the ACC was indeed indirect, as the OXT receptor antagonist Ato prevented the increase in LTP. This implies that the effect of MDMA was mediated by OXT released in the ACC, downstream of MDMA-induced non-exocytotic 5-HT release. The best to our knowledge, there are still no available data indicating the existence of presynaptic 5-HT heteroreceptors on OXT-containing axon terminals or reporting a local modulator activity of 5-HT on OXT release in the cerebral cortex of mammals. However, and considering that DRN is the main source of 5-HT for the hypothalamus (Muzerelle et al., 2016), Liu et al. (2023) have shown that OXT neurones in the PVN are structurally, electrophysiologically, and functionally downstream of 5-HT neurones of the DRN. This may suggest that 5-HT may serve as a key regulator of OXT neurones in social behaviours. It seems then likely that MDMA increases 5-HT levels in the PVN, stimulating OXT release in several brain targets, including the ACC, thereby modulating various prosocial behaviours, as noted above. This view is supported by the fact that in the present study, intra-PVN microinjection of Ketan prevented the LTP increase in the ACC generated by MDMA, which implies the involvement of 5-HT_2A_ receptors located in PVN OXT neurones in the downstream communication with the ACC.

OXT acting in ACC mediates empathy-based consoling behaviours in prairie voles (Burkett et al., 2016) and many of the prosocial effects of MDMA have been shown to be ultimately mediated by OXT (Thompson et al., 2007; Ramos et al., 2013), including helping behaviour (Yamagishi et al., 2020). In addition, it is known that 5-HT stimulates hypothalamic paraventricular OXT-containing neurones via 5-HT_1A/2A_ receptors, which leads to increased OXT release in several brain regions, including the ACC (Jurek and Neumann, 2018; Arakawa, 2021), thereby supporting a role for OXT mediation of ACC LTP downstream of 5-HT signalling. It then appears that, hypothetically, the LTP process we observed in the ACC might be important for the prosocial effects of MDMA and, probably, for the motivational drive of helping behaviour. Indeed, as noted previously, there are studies claiming functional associations between neural plasticity undergoing some specific neurones of the ACC and the occurrence of action execution: (i) the ACC contains distinct neuronal populations that separately control consoling allolicking and allogrooming behaviour (Zhang M. et al., 2024); (ii) emotional mirror neurons in the ACC of helper rodents are recruited in response to witnessing a conspecifics distress (Carrillo et al., 2019; Wu and Hong, 2022; Wu et al., 2022); (iii) activity in certain ACC neurons of helper rats changes dynamically during helping behaviour (Song et al., 2023); (iv) a subpopulation of neurons projecting from the ACC to the NAc is positively correlated with the percent of door-openings across testing sessions in helping behaviour (Ben-Ami Bartal et al., 2021); (v) OXT receptor antagonism in the ACC reduces comforting in prairie voles (Burkett et al., 2016); and (vi) targeted helping behaviour is modulated by OXT signalling in the ACC (Yamagishi et al., 2020). Although these studies highlight the role of the ACC in prosocial consoling and targeted helping actions in rodents, further research is needed to clarify whether the OXT-dependent LTP-like neuroplasticity that occurs in the rat ACC is required to achieve the prosocial effects induced by MDMA, as well as for the motivational drive underlying helping behaviour.

The results obtained in the present work could be of clinical interest. MDMA is known to promote both prosocial behaviour and emotional empathy (Hysek et al., 2014), and its therapeutic potential is diverse (Dunlap et al., 2018). In general, the effects associated with the entactogenic syndrome produce pro-empathetic behavioural effects in humans that are clinically useful (Wolfgang et al., 2025), which are usually associated with non-chronic use of the drug. In contrast, habitual consumption of MDMA can diminish pro-empathetic effects and increase the occurrence of toxic effects (Peroutka et al., 1988), which can disrupt individuals’ prosocial abilities. One could speculate about the possibility that repeated exposure to MDMA induces an individual-level disability, which would be expressed by the dissociation between the ability or interest to perform a prosocial behaviour and its effective execution.

In conclusion, the results obtained in the present work showed that the administration of subtoxic doses of MDMA disrupt helping behaviour. In contrast, the neuroplastic effects elicited by MDMA in the NAc and ACC are quite aligned with those 5-HT-mediated mechanisms that promote prosocial/empathic behaviour, suggesting that the increased neuroplasticity observed (likely related to a greater motivational drive underlying helping behaviour) remains unaffected. Therefore, it is proposed that the disruption of helping behaviour induced by MDMA might be due to a maladaptive regulation of the learning or memory processes required to acquire the knowledge or to remember how to open the door to free the soaked animal. More research is needed to further elucidate how MDMA may alter brain circuits involved in performing helping behaviours and processing the distress of the soaked cagemate.

## Data Availability

The raw data supporting the conclusions of this article will be made available by the authors, without undue reservation.
